# Identification of Novel Reference Genes Based on MeSH Categories

**DOI:** 10.1371/journal.pone.0093341

**Published:** 2014-03-28

**Authors:** Tulin Ersahin, Levent Carkacioglu, Tolga Can, Ozlen Konu, Volkan Atalay, Rengul Cetin-Atalay

**Affiliations:** 1 Department of Molecular Biology and Genetics, Bilkent University, Ankara, Turkey; 2 Computer Engineering Department, Middle East Technical University, Ankara, Turkey; Nazarbayev University, Kazakhstan

## Abstract

Transcriptome experiments are performed to assess protein abundance through mRNA expression analysis. Expression levels of genes vary depending on the experimental conditions and the cell response. Transcriptome data must be diverse and yet comparable in reference to stably expressed genes, even if they are generated from different experiments on the same biological context from various laboratories. In this study, expression patterns of 9090 microarray samples grouped into 381 NCBI-GEO datasets were investigated to identify novel candidate reference genes using randomizations and Receiver Operating Characteristic (ROC) curves. The analysis demonstrated that cell type specific reference gene sets display less variability than a united set for all tissues. Therefore, constitutively and stably expressed, origin specific novel reference gene sets were identified based on their coefficient of variation and percentage of occurrence in all GEO datasets, which were classified using Medical Subject Headings (MeSH). A large number of MeSH grouped reference gene lists are presented as novel tissue specific reference gene lists. The most commonly observed 17 genes in these sets were compared for their expression in 8 hepatocellular, 5 breast and 3 colon carcinoma cells by RT-qPCR to verify tissue specificity. Indeed, commonly used housekeeping genes *GAPDH*, *Actin* and *EEF2* had tissue specific variations, whereas several ribosomal genes were among the most stably expressed genes *in vitro*. Our results confirm that two or more reference genes should be used in combination for differential expression analysis of large-scale data obtained from microarray or next generation sequencing studies. Therefore context dependent reference gene sets, as presented in this study, are required for normalization of expression data from diverse technological backgrounds.

## Introduction

During the last decade, there has been remarkable progress in the identification of human cells' transcriptome blueprint through small or large-scale quantitative gene expression studies. Since the gene expression is the major determinant of the protein abundance in the cell, transcriptome analysis experiments have been widely applied to reveal the molecular mechanisms of various cellular conditions. Depending on the cell fate, expression levels of genes vary. Although it seems straightforward to assess these variations through Real Time quantitative PCR or microarrays, there is a continuing and confusing debate on what basis these variations should be considered as deviations from normal physiology. Therefore, there is a need for compilation and comprehensive analysis of a gene of interest across several experiments from different sources. A gene's expression data must be scaled in a comparable platform. Various normalization methods are available to scale these data within the same experiment, yet it becomes problematic to compare arrays of different sources without using references.

Although most genes show variable expression depending on cellular context, tissue of origin or treatment conditions, some genes are constitutively expressed in all cells in all conditions. These constitutively expressed genes are required for the maintenance of the basal cellular functions such as metabolism, gene expression, protein synthesis and cell signaling [1], [2]. These genes, called housekeeping genes, are generally assumed to have expression levels unaffected by tissue of origin or experimental condition. Therefore, they are widely used as reference genes for normalization of expression data. However, recent studies indicated that several widely used housekeeping genes have altered expressions under different experimental conditions [3]–[10]. Most of these studies focused on finding appropriate genes for normalization in individual cancer types. Yet, even the most commonly used reference genes (*ACTB, GAPDH, TBP*) were differentially expressed in different pathological stages of hepatocellular carcinoma [7], [11]. These findings bring forward the need to process high-throughput data in order to determine a global list of constitutively and invariably expressed genes that can be used as reference genes [12]–[14]. For example, Hruz T. et al. measured the standard deviation of gene expression across large sets of Affymetrix arrays of human, mouse and *Arabidopsis* from the Genevestigator database and developed an online tool, Ref-Genes, that can be used to search for genes with minimal standard deviation across a chosen set of arrays [15], [16]. They concluded that no genes are universally stable, but a subset of stable genes with minimal variance exists for each biological context that can be used for the normalization of RT-qPCR data.

Although publicly available microarray or next generation sequencing (NGS) experiments were used to generate lists of candidate reference genes, novel statistical approaches for testing accuracy of a reference gene are still needed. Herein, we aimed to confirm the reliability of available housekeeping gene sets using randomization as well as to determine other invariably expressed gene sets based on Receiver Operating Characteristic (ROC) curves for classifiers under large number of experimental conditions and across a wide panel of tissue types.

Our method provides reference gene lists for global and cell-type specific normalization of transcriptome data. Gene lists are scored based on their expression stability, and classified according to the Medical Subject Headings (MeSH) associated with the transcriptome study that was published and indexed by National Library of Medicine. Gene lists are provided in the supporting dataset (Supporting Information S1). RT-qPCR assessment of selected reference genes is also provided for various tissue-specific cancer cell lines *in vitro*.

## Results and Discussion

### Development of a methodology to identify consistently stable genes

Housekeeping/reference genes should exhibit relatively constant expression levels when compared to non-housekeeping genes. To identify the consistency of the so-called housekeeping genes across large-scale experiment sets, the gene expression data were downloaded from the NCBI Gene Expression Omnibus (NCBI-GEO) database [17], and all spot data were extracted along with their associated metadata from the platform files. The data set approximately contained 142 million oligonucleotide microarray spots from 9090 microarray samples, which were grouped into 381 GEO datasets. Percentile-ranking method was applied independently on the global mean normalized data within each sample in each GEO dataset. This process provided a rank value for each gene within a sample. Therefore, the rank measure was comparable across experiments and platforms, allowing the analysis of the behavior of a gene globally across GEO datasets. Using ranks of genes is a standard method as also employed in Quantile Normalization, which is a common microarray normalization technique [18].

**Figure 1:**
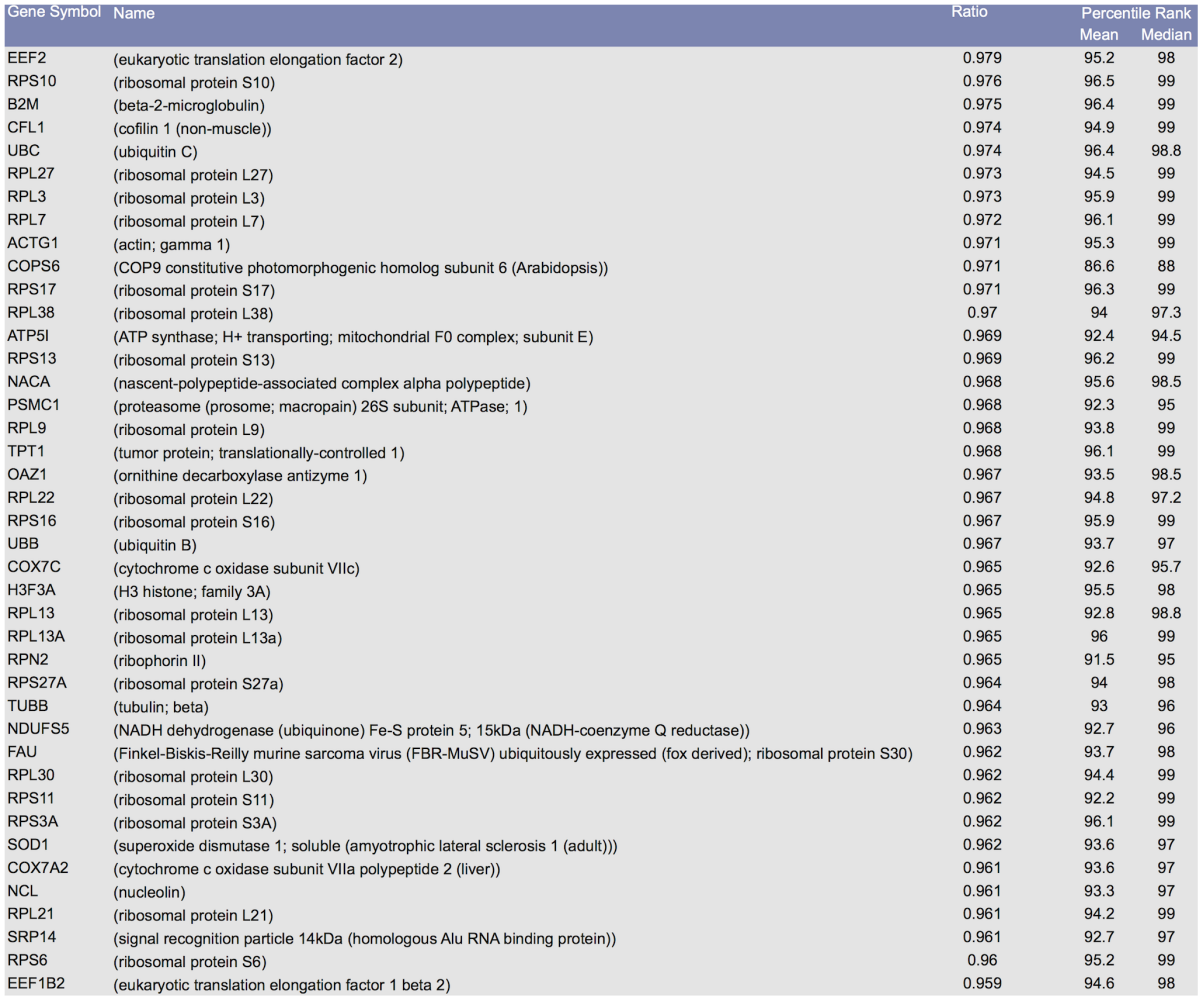
Screenshot of the first 40 lines from reference gene lists. Complete set of MeSH classified reference gene lists from 9090 array samples are given in hyperlinked Supporting Information S1 spreadsheet.

The average changes in the rank of each gene in each GEO dataset (GDS) was computed based on the ratio of the standard deviation to the mean and termed as its coefficient of variation value (CV) (see Methods). CV, as a standardized measure of sample variability, help suggest candidate reference genes whose expression is stable and unaffected by the experimental condition since it has been successfully applied in identification of reference genes in multiple studies with varying thresholds [19]–[22]. Most genes had coefficient of variation values only for a subset of the available GDSs. Therefore, for each gene *G_i_* and a predefined CV threshold *t*, the ratio of GEO datasets, where CV is less than *t*, Ratio_t_(*G_i_*), is calculated as the measure of stable expression. Ratio_t_(*G_i_*) is the ratio of GEO datasets, in which a gene exhibits a coefficient of variation less than *t*. The Ratio_t_(*G_i_*) value, by itself, is not a sufficient measure to identify statistically significant reference genes. A gene that has a small enough CV value can get a perfect ratio even if it is observed in only a single GEO dataset. Therefore, a new parameter, PO(*G_i_*), calculated as the percentage of datasets that each gene has been observed in at least once, was used to adjust Ratio_t_(*G_i_*) parameter (Methods). Ratio_t_(*G_i_*) in the context of PO(*G_i_*) allowed for accurate normalization of a large set of microarray data, since it takes into account the information about the differences in probe/clone composition of the arrays. We assessed the utility of these measures by implementing a simple threshold based classifier and computing the sensitivity and specificity of this classifier using a published reference gene set as the ground truth (Methods). Two sets of reference genes were generated. First list contains reference gene lists with a CV threshold of 0.12 and with various sensitivity values while the second list is built with sensitivity of 0.5 for a range of CV threshold values. Figure 1 shows the first 40 genes from 342 reference genes with a CV of at most 0.12, sensitivity equal or over 0.5, specificity of 0.97 and minimum percentage of occurrence of 0.75 in all 9090 array samples from 381 GEO datasets (See Reference Gene Lists in Supporting Information S1 for the complete list). The high specificity shows that the CV measure coupled with percentage of occurrence is an accurate measure for identification of reference gene sets.

Housekeeping/Reference genes should exhibit relatively constant expression levels and their average rank change should be lower than that of remaining genes. Hence, we assumed that the candidate reference genes should have lower CV and higher Ratio_t_(*G_i_*) than that of randomly selected genes. In order to compare the expression behaviors of reference genes to that of random genes, we first analyzed the largest previously reported housekeeping datasets [1], [23]. There was high variation between genes in the first dataset, in parallel with the authors' observations. Therefore, the dataset provided by Eisenberg *et al.* was used in our analysis. The 566 housekeeping genes in this dataset were compared to five different randomly selected sets of non-housekeeping genes having the same mean rank distribution as that of the housekeeping gene set.

**Figure 2:**
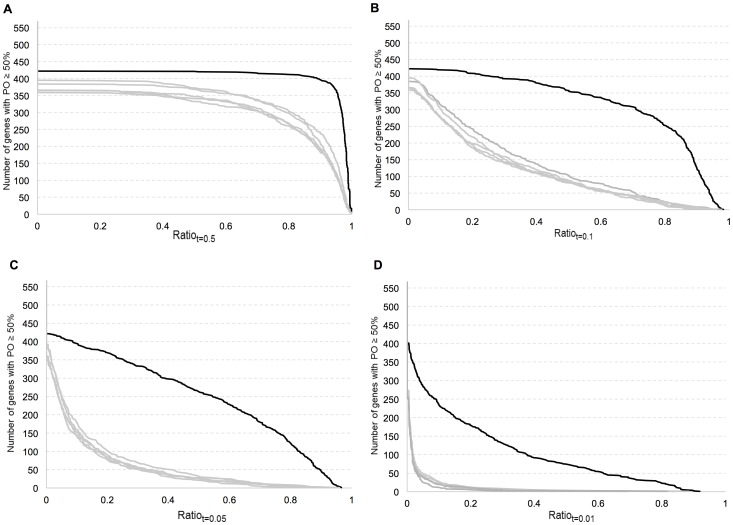
Graph of ratio of the number of sets in which a gene has a coefficient of variation (CV) less than a threshold (t), to the number of sets in which the gene is observed. Graphs were plotted for CV value thresholds t = 0.5 t = 0.1, t = 0.05 and t = 0.01. Percentage of occurrence (PO) is at least 50% of the total sets. The y-axis indicates the number of genes having a ratio greater than the ratio value at the corresponding x-axis. This function is described in the methods section, as x-axis being r and y-axis being f_PO_(r). The black curve represents housekeeping genes while curves with grey colors show 5 random sets of genes excluding the housekeeping genes. Random sets of genes have the same mean rank distribution as of those housekeeping genes.

**Figure 3:**
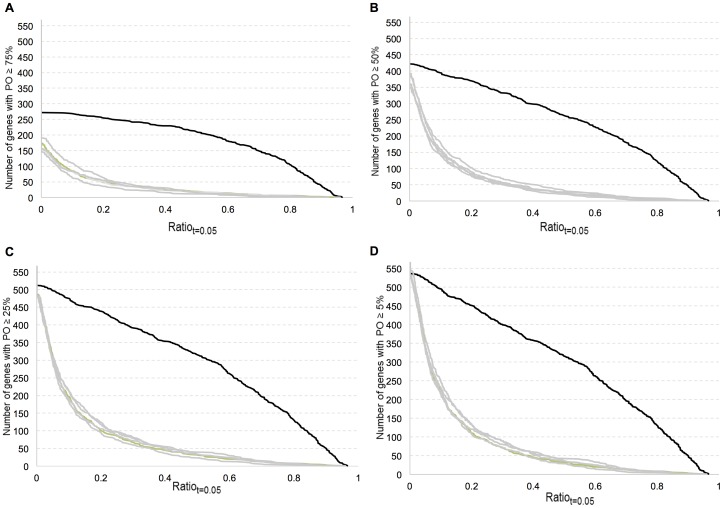
Graph of ratio of the number of sets in which gene has coefficient of variation less than 0.05 to the number of sets in which the gene is observed. Gene is observed at least a-) 75%, b-) 50%, c-) 25% and d-) 5% of the total sets. The y axis indicates the number of genes having a ratio greater than ratio value at the corresponding x axis. The curve with red color represents housekeeping genes while curves with other colors shows 5 random sets of genes excluding the housekeeping genes. Random sets of genes have the same mean rank distribution as of those housekeeping genes.

Normalized gene expression values were analyzed for the CV thresholds *t* = 0.5, *t* = 0.1, *t* = 0.05, and *t* = 0.01 and minimum percentage of occurrences PO = 75%, 50%, 25% and 5%. When coefficient of variation, CV, was less than 0.5 (*t* = 0.5), percentile-ranked GEO datasets showed high Ratio*_t_* values for nearly all of the analyzed genes (housekeeping or not), for all PO values. At lower CV thresholds, *t* = 0.1, *t* = 0.05 and *t* = 0.01, housekeeping genes had significantly higher Ratio*_t_* values than those of random gene sets for all PO values. The randomization approach allowed us to test an optimum range of *t* and PO values that can discriminate between reference and non-reference genes. Graphs of Ratio*_t_* at PO = 50% with four different CV thresholds (Figure 2) and graphs of Ratio*_t_* at CV *t* = 0.05 with four different PO thresholds were plotted (Figure 3). These graphs showed that measures could accurately distinguish previously identified reference genes from the randomly selected ones.

The difference in the ratio distribution of housekeeping genes compared to that of the randomly selected non-housekeeping gene sets was statistically significant, as shown by Kolmogorov-Simirnov tests (Table S1 in Supporting Information S2, p<0.0001). The random gene sets, excluding the housekeeping genes, did not show any significant ratio distribution difference, as shown by using Bonferroni adjusted Kolmogorov-Simirnov tests (Table S2 in Supporting Information S2). These observations proved that the expression of the tested housekeeping genes was less variable across different experiment sets compared to that of randomly selected gene sets.

**Figure 4:**
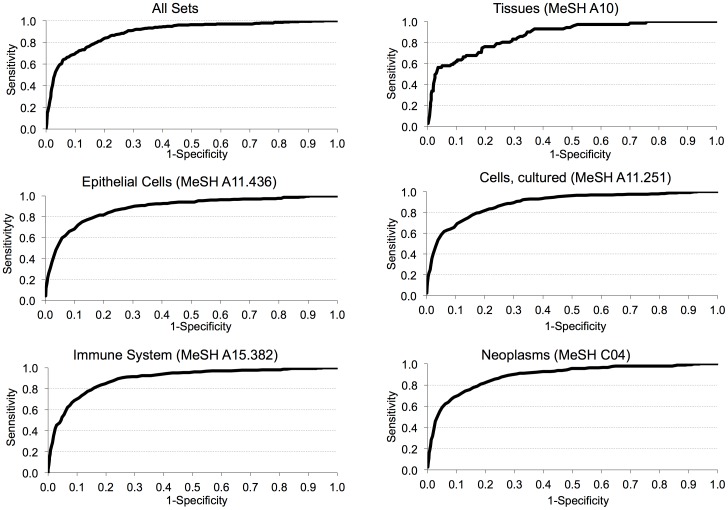
Receiver Operator Characteristic (ROC) curve of the simple threshold based classifier. The receiver operator characteristic (ROC) curve of a simple threshold classifier over all datasets and some MeSH categories. The housekeeping gene set by Eisenberg et al. [22] is used as the ground truth. The simple threshold classifier classifies all the genes with CV values below a threshold as housekeeping genes. By using different CV thresholds the stringency of the classifier can be varied and the ROC curve can be plotted accordingly. Sensitivity is the ratio of correctly classified ground truth genes over all ground truth genes and specificity is the ratio of correctly identified non-housekeeping genes over all non-housekeeping genes. Complete set of MeSH classified RO-curves are given in hyperlinked Supporting Information S3 spreadsheet.

In addition, we assessed the sensitivity and specificity of a simple threshold based classifier using the CV threshold using the published housekeeping gene set as the ground truth. The receiver operator characteristic (ROC) curve in Figure 4 shows that more than half of the published reference genes can be identified with a specificity of 0.97. Similar analyses performed on datasets grouped by MeSH are available in Supporting Information S3.

### Identification of novel reference genes

Our primary goal in this study was to define a novel reference gene set that can be used for both global and cell type-specific normalization of expression experiments. For this purpose, a classifier that can be used to identify novel reference genes was built. Based on our analysis with the known 566-housekeeping gene set, coefficient of variation (CV) measure was set as the variable for building the classifier while minimum percentage of occurrence (PO) and Ratio*_t_* values were fixed at 75% and 0.90 respectively. The accuracy of this classifier in predicting reference genes was assessed in comparison with the previously reported 566 housekeeping gene set [23]. Among the candidate reference genes that were identified by our classifier at each CV threshold, the known 566 housekeeping genes were regarded as true positives (TP) and the other genes were regarded as false positives (FP) to plot a receiver-operating characteristic (ROC) curve. In the supporting gene lists, the sensitivity was set to 0.5, implying that half of the identified reference genes are the known housekeeping genes. The ROC curve of our classifier for CV values ranging from 0.01 to 10 showed its effectiveness in finding true positives (sensitivity) (Figure 4). Curves for the overall (Figure 4A) and specific reference gene sets are available in the supporting hyperlinked dataset (Supporting Information S3).

According to a classic ROC curve, a good classifier should capture most of the known housekeeping genes while providing a relatively small number of false positives. However, in this particular case, the false positives could be the newly identified candidate reference genes. Therefore, their CV and *Ratio_t_* values should still be considered for their potential as a reference gene. CV, Ratio and Percentile Rank values are provided for each gene in the supporting hyperlinked dataset. The global reference genes were given in this list under the category of *All* with CV of 0.12, sensitivity equal or over 0.5, specificity of 0.97 and minimum percentage of occurrence of 0.75 (Figure 2A and Supporting Information S1).

In order to determine origin- and cell type-specific reference genes, the GEO sets were classified according to the Medical Subject Headings (MeSH) associated with their experimental data, published and indexed by National Library of Medicine. Of the 381 GEO datasets analyzed in this study, 341 were associated with 272 different medical publications and 264 of these publications were associated with a total of 5754 MeSH terms [24]. These gene sets were grouped into three anatomy (tissues, cells, hemic and immune systems), and one disease (neoplasms) based MeSH categories (Table 1). GDS identification numbers associated with each MeSH are given in Supporting Information S1. Two lists of reference gene sets based on CV and sensitivity, were provided for each MeSH category. First list was constructed based on a fixed threshold for CV<0.12 and the second on a fixed threshold of sensitivity>0.50. Both gene sets had a fixed PO threshold of 75%. The reference gene lists, which include CV, PO, specificity and sensitivity values for each MeSH category, are provided as supporting hyperlinked dataset (Supporting Information S1).

**Table 1:** MeSH groups and number of NCBI-GEO data sets in each group.

MeSH Tree Number	MeSH Heading	Number of Sets
ALL	ALL	381
A10	Tissues	77
A10.272	Epithelium	16
A10.690	Muscles	46
A11	Cells	223
A11.118	Blood Cells	47
A11.148	Bone Marrow Cells	14
A11.251	Cells. Cultured	157
A11.284	Cellular Structures	40
A11.329	Connective Tissue Cells	34
A11.436	Epithelial Cells	48
A11.627	Myeloid Cells	17
A11.733	Phagocytes	14
A11.872	Stem Cells	22
A15	Hemic and Immune Systems	74
A15.145	Blood	51
A15.378	Hematopoietic System	14
A15.382	Immune System	60
C04	Neoplasms	108
C04.557	Neoplasms by Histologic Type	68
C04.588	Neoplasms by Site	68
C04.697	Neoplastic Processes	16

### Experimental validation of selected reference genes in different cancer cells

**Figure 5:**
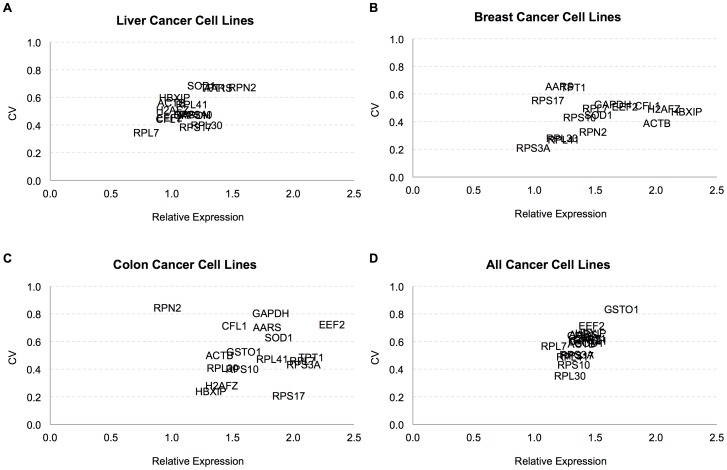
Graphs of coefficient of variations and relative expression levels of 17 reference genes in RT-qPCR. Coefficient of Variation (CV) was calculated based on the relative expression (efficiency^−ΔCq^) of each housekeeping gene in (**A**) Liver, (**B**) Breast, (**C**) Colon and (**D**) All cancer cell lines.

Among the large panel of identified reference genes, 17 genes were selected for experimental validation (Table 2). Expression levels of these reference genes (*AARS, ACTB, CFL1, EEF2, GAPDH, GSTO1, H2AFZ, HBXIP, RPL30, RPL41, RPL7, RPN2, RPS10, RPS17, RPS3A, SOD1, TPT1*) were assessed by RT-qPCR in 16 different cell lines consisting of 8 Hepatocellular Carcinoma (HCC) (HepG2, FOCUS, Mahlavu, Hep3B, Hep3B-TR, Huh7, SkHep1, and PLC), 5 Breast Cancer (MDA-MB453, HCC1937, BT20, T47D and CAMA-I), and 3 Colon Cancer (HCT116, HT29, and SW620) cell lines. Housekeeping/reference genes are expected to have high expression and low variability in expression levels between cells. Therefore, in RT-qPCR amplification, they should have low threshold cycle (Cq <30) and low standard deviation. All of the tested reference genes met this requirement for Cq values and standard deviations (Figure 5). The best candidate reference genes appeared at the center of the graphs for each group (Figure 5 A–C). In order to emphasize the stability of these genes, a well-expressed non-housekeeping gene, *RECK*, was included into the CV analysis. It was not included in the analysis with NormFinder and geNorm due to its high variability (CV around 1.0). Genes were ranked by their stability based on their CV. Comparison of the expression values by analysis of variance (ANOVA) identified *RPL30* as the most stable gene with lowest variance within and between the Liver, Breast and Colon Carcinoma cell line groups, followed by *RPL41*, *RPS10* and *CFL1*. The variance within each of these three groups was low for *RPS3A, RPS17, RPL7, ACTB, H2AFZ*, and *HBXIP* reference gene expression. However, the variance between the three groups was relatively high. This implied that these reference genes are more suitable for normalization of cell lines from single tissue-origin than cell lines with different tissue-origins. *GSTO1, TPT1, RPN2, SOD1* showed the highest variability.

**Table 2:** Stability measures of the reference genes that were used in RT-qPCR analysis.

	All 381 GEO sets			MeSH A11.251 (cells,cultured)			MeSH C04 (Neoplasms)		
		Percentile Rank			Percentile Rank			Percentile Rank	
Gene Symbol *	Ratio	Mean	Median	Ratio	Mean	Median	Ratio	Mean	Median
**RPS3A**	1	96.1	99	1	94.7	99	1	97.3	99
**RPL30**	1	94.4	99	1	95.6	99	1	98.7	99
**RPS10**	1	96.5	99	1	95.4	99	1	98.2	99
**RPL7**	1	96.1	99	1	95	99	1	98	99
**RPL41**	0.9	93.8	99	0.9	92.2	99	0.9	96.7	99
**CFL1**	1	94.9	99	1	93.7	99	1	97.8	99
**RPS17**	1	96.3	99	1	94.7	99	1	97.7	99
**H2AFZ**	0.9	90.7	94	1	92.4	86	0.9	93.5	95
**ACTB**	0.9	95	99	1	95.3	99	1	98.7	99
**HBXIP**	1	89	92	1	88.7	92.5	1	90.4	92
**EEF2**	1	95.2	98	1	93.5	98.5	1	97	98.5
**AARS**	0.9	85.7	91	0.9	87.2	92	0.9	87.2	92
**SOD1**	1	93.6	97	1	92.2	97	1	95	97
**TPT1**	1	96.1	99	1	95.3	99	1	97.7	99
**RPN2**	1	91.5	95	1	91.4	96	1	94	96.7
**GSTO1**	0.9	90	94	0.9	89.4	94	0.9	90	93

*Selected genes with CV ≤0.12 and PO ≥75%. The complete results for all MeSH categories and all reference genes obtained from 381 GEO sets are given in the Supporting Reference Gene Lists dataset (Supporting Information S1).

**Figure 6:**
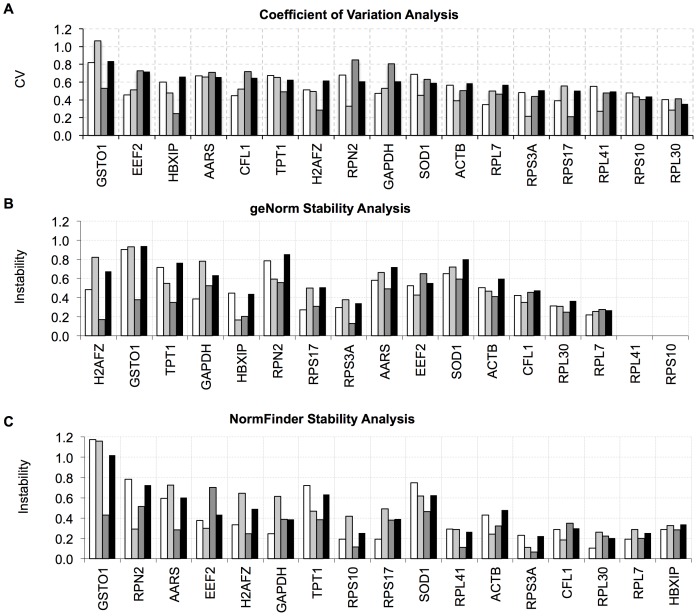
Stability analysis of reference genes in RT-qPCR based on CV, geNorm and NormFinder. Genes ranked by stability based on (**A**) CV, (**B**) geNorm and (**C**) NormFinder tools. White, light gray, dark gray and black bars represent Liver, Breast, Colon and All Cancer Cell lines respectively.

**Figure 7:**
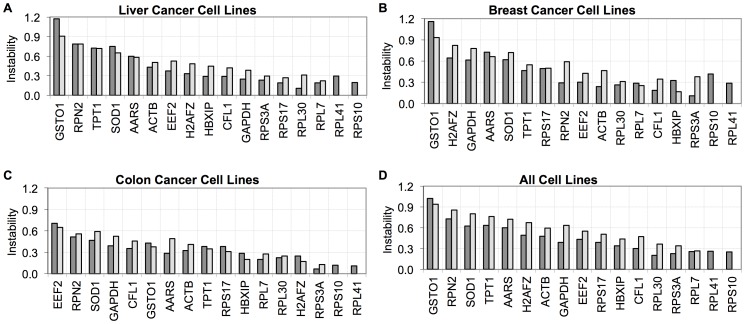
Stability analysis of reference genes in tissue-specific cell lines in RT-qPCR. Stability analysis in (A) Liver, (B) Breast, (C) Colon and (D) All cancer cell lines. NormFinder and geNorm results were represented in dark gray and light gray respectively.

Real-time quantitative PCR gene expression stability was further determined using geNorm and NormFinder software [3], [25]. GeNorm is a pairwise comparison-based model. For each gene, it calculates an expression stability value based on the average pairwise variation between all tested genes. The genes are ranked according to their expression stability through stepwise exclusion of the least stable gene (highest stability value). NormFinder is a model-based approach that estimates the variation between sample subgroups, such as liver, breast and colon cancer cell lines, as well as the overall expression variation of the tested genes. Unlike geNorm, the resulting stability value and the stability rank order changes in NormFinder depending on the input genes. Therefore, geNorm and NormFinder stability analysis was performed with and without the least stable genes *EEF2, GSTO1, RPN2* and *TPT1*, which have a standard deviation value above the mean standard deviation (stdev = 1.42). The selected reference genes were ranked according to their overall stability values across 16 different cell lines, determined by geNorm and NormFinder (Table 3, Figure 6, Table S3 in Supporting Information S2). The ranking of these genes was similar for both methods. Ribosomal protein genes *RPS10, RPL41, RPL30*, and *RPS3A* were the most stable genes among all 16 cell lines according to geNorm and NormFinder respectively (Table 3, Tables S3–S4 in Supporting Information S2). Two traditional reference genes, *ACTB* and *GAPDH*, were less stable than the ribosomal genes and ranked lower in the stability rank list. *CFL1* and *HBXIP* were the most stable genes after the ribosomal protein genes in general (Figure 7A and Table 3). While the commonly used reference gene *GAPDH* was one of the stable genes within liver cancer cell lines, it was among the least stable genes within breast and colon cancer cell lines. It had a high variance in terms of stability value and expression value between the three types of carcinoma cell lines investigated. This implied that *GAPDH* could be used as a reference gene when comparing liver cancer cell lines, but not breast and colon cancer cell lines. *ACTB* was more stable than *GAPDH* in Breast and Colon Carcinoma cell lines and had a stability value similar to *GAPDH* in HCC cell lines. Moreover, even though reference genes were known to avoid regulation by miRNAs, recent findings showed that *GAPDH* and *ACTB* are direct targets of miR-644a [26], [27]. Besides, several pseudogenes of *GAPDH* and *ACTB* were revealed, making them less reliable to be used as reference genes [28].

**Table 3:** Stability order of 17 housekeeping genes based on CV, NormFinder and geNorm analysis.

All			Liver			Breast			Colon		
CV	NormFinder	geNorm	CV	NormFinder	geNorm	CV	NormFinder	geNorm	CV	NormFinder	geNorm
RPL30	RPL30	RPL41	RPL7	RPL30	RPL41	RPS3A	RPS3A	RPL41	RPS17	RPS3A	RPL41
RPS10	RPS3A	RPS10	RPS17	RPL7	RPS10	RPL41	CFL1	RPS10	HBXIP	RPL41	RPS10
RPL41	RPS10	RPL7	RPL30	RPS17	RPL7	RPL30	ACTB	HBXIP	H2AFZ	RPS10	RPS3A
RPS17	RPL7	RPS3A	CFL1	RPS10	RPS17	RPN2	RPL30	RPL7	RPS10	RPL7	H2AFZ
RPS3A	RPL41	RPL30	EEF2	RPS3A	RPS3A	ACTB	RPL7	RPL30	RPL30	RPL30	HBXIP
RPL7	CFL1	HBXIP	GAPDH	GAPDH	RPL30	RPS10	RPL41	CFL1	RPS3A	H2AFZ	RPL30
ACTB	HBXIP	CFL1	RPS10	CFL1	GAPDH	SOD1	RPN2	RPS3A	RPL7	AARS	RPL7
SOD1	GAPDH	RPS17	RPS3A	HBXIP	CFL1	HBXIP	EEF2	EEF2	RPL41	HBXIP	RPS17
GAPDH	RPS17	EEF2	H2AFZ	RPL41	HBXIP	H2AFZ	HBXIP	ACTB	TPT1	ACTB	TPT1
RPN2	EEF2	ACTB	RPL41	H2AFZ	H2AFZ	RPL7	RPS10	RPS17	ACTB	CFL1	GSTO1
H2AFZ	ACTB	GAPDH	ACTB	EEF2	ACTB	EEF2	TPT1	TPT1	GSTO1	RPS17	ACTB
TPT1	H2AFZ	H2AFZ	HBXIP	ACTB	EEF2	CFL1	RPS17	RPN2	SOD1	TPT1	CFL1
CFL1	AARS	AARS	AARS	AARS	AARS	GAPDH	GAPDH	AARS	AARS	GAPDH	AARS
AARS	SOD1	TPT1	TPT1	TPT1	SOD1	RPS17	SOD1	SOD1	CFL1	GSTO1	GAPDH
HBXIP	TPT1	SOD1	RPN2	SOD1	TPT1	TPT1	H2AFZ	GAPDH	EEF2	SOD1	RPN2
EEF2	RPN2	RPN2	SOD1	RPN2	RPN2	AARS	AARS	H2AFZ	GAPDH	RPN2	SOD1
GSTO1	GSTO1	GSTO1	GSTO1	GSTO1	GSTO1	GSTO1	GSTO1	GSTO1	RPN2	EEF2	EEF2

Stability within liver, breast and colon cancer cell lines was also analyzed separately. The ribosomal genes *RPL30, RPL41, RPL7, RPS10*, and *RPS3A* were stable within each cell line group (Figure 7B–D, Table 3, Tables S3, S4 in Supporting Information S2). *HBXIP*, *CFL1*, and *GAPDH* were among the most stable genes together with the ribosomal protein genes in 8 HCC cell lines analyzed. *HBXIP* was the third most stable gene in breast cancer cell lines according to geNorm, but ninth according to NormFinder ranking. *H2AFZ* and *HBXIP* were ranked among the most stable genes together with *RPS10*, *RPL41*, and *RPS3A* in colon cancer cell lines. The difference in the gene stability ranking order between the two softwares is due to the different methodologies. geNorm is based on the comparison of expression similarity of the tested genes. This may cause exclusion of a candidate reference gene with a relatively stable expression in early steps of the ranking procedure, if all other genes in the list have similar expression profiles. Another disadvantage of this approach may be a bias towards co-regulated genes, since these genes will have similar expression profiles and hence may be ranked top in the stability list, regardless of their expression stability. NormFinder does not have such a bias, since expression stability of each gene is determined independent of the rest of the genes. However, since NormFinder ranking is based on the variation between groups, when comparing groups like liver, breast, and colon carcinoma cell lines, if the variation within group is high, then the variation between groups may considered as lower, leading to false positive results.

In order to determine the optimal number of reference genes for accurate normalization, pairwise variation V_n/n+1_ analysis was performed using the geNorm software. Taking 0.15 as a cut-off value as proposed, the use of *RPL41* and *RPS10* together as reference genes were enough for accurate normalization (V2/3 value = 0.134) when comparing Liver, Breast and Colon Carcinoma cell lines. An even more accurate normalization can be achieved if *RPL7* and *RPS3A* are also included as reference genes. (V3/4 value = 0.091 and V4/5 value = 0.067).

## Conclusions

Even the most frequently used reference genes are subject to differential regulation under specific treatments or between different cell lines or tissues. Therefore, new reference gene sets should be determined instead of using traditional housekeeping/reference genes that are themselves prone to differential regulation. The use of two or more housekeeping genes for normalization can improve the reliability of normalization [5], [9], [29]. The largest meta-analysis for reference gene identification up-to-date compiled 1431 samples from 104 microarray data sets classified into 4 physiological states with 13 organ/tissue types and identified reference gene candidates mostly associated with transcription, RNA processing and translation [30]. Hruz et al. 2011 also performed a large scale meta-analysis across multiple species and sources using Genevestigator database [16]. They have used ranks of standard deviations using mouse Affymetrix datasets to support context specificity of reference gene sets. Our study also is comprehensive containing 142 million oligonucleotide microarray spots from 9090 microarray samples, grouped into 381 GEO datasets from multiple platforms. We also provide a novel methodology based on randomizations and ROC that allows testing the specificity and sensitivity of classifying a gene as reference or non-reference. Previously, a meta-analysis of 13,629 human gene array samples from GEO database identified candidate housekeeping genes, including *RPS13*, *RPL27, RPS20* and *OAZ1*. For each gene the coefficient of variation (CV) of its expression and the maximum fold change was calculated to identify genes with the minor variation in expression [19]. Recently, Eisenberg *et. al*. published an updated new housekeeping gene list based on analysis of RNA-seq data [31]. However, MeSH classification used in the present study has not been applied to reference gene set combinations previously. In this study, we determined novel reference gene sets that can be used for both global and MeSH category-based normalization of gene expression data. Furthermore, we validated stability of known and novel reference genes obtained from meta-analysis with cancer cell line qPCR studies.

Our reference gene lists were dominated by the ribosomal protein genes and genes that are involved in maintenance of basal cellular activities such as translation and metabolism. Especially, *RPL30* and *RPL41* are good reference genes for comparing all cell lines regardless of their origin. Previously, 451 housekeeping genes that were expressed in all of the 19 distinct normal human tissue types studied, were shown to have variable expression levels among different tissues and ribosomal genes, which were among the most stable genes, were suggested as reference genes suitable for normalization purposes [1]. A more recent study however suggested that ribosomal protein genes, which display stable expression in meta-analysis, indeed exhibit variation in mRNA expression in a tissue-dependent manner [32]. These findings once more emphasize that finding a common reference gene, ribosomal or not, is not possible. The genes located at the top the reference gene set lists display the highest confidence based on CV and PO values therefore those genes are less likely to be wrong. However every computational analysis might have false positive results for this reason, users should select their reference gene of interest and experimentally validate the stability of the expression of that reference gene under their experimental conditions. Furthermore more than 2 reference genes per experiment would be a better measure for precision. Computational calculations present a road map to guide the experimentalists. The data sets we used to build MeSH dependent reference gene lists originated from various sources which aimed to identify differentially expressed gene sets under specific experimental conditions in order to minimize the homogeneity between and within data sets. Data set IDs are given together with reference gene lists in Supporting Information S1. Meta-analysis and consequent classification of tissue- or cell type- origin specificity of housekeeping genes appears to be the best approach to determine the most appropriate reference genes among the large number of known housekeeping genes. Furthermore identification of housekeeping genes enables normalization of transcriptome data not only for microarrays but also for RNA-seq experiments [33], [34]. RNA-seq technology is advantageous for detecting genes that are expressed in low levels. Hence, similar studies with RNA-seq data may increase the reliability of housekeeping genes when large number of NGS data are available [14] and can be used to identify a universal reference gene set. The tissue specific reference gene lists presented in this study, provide housekeeping genes that can be exploited as references in differential expression analysis of data from variety of transcriptome and RT-qPCR experiments.

## Methods

In order to normalize expression values, percentile ranking have been applied. For each gene *G_i_* in a sample *e_i_*, a single rank value, *r*(*G_i_*.*e_j_)* was computed (Equation 1). For a gene that was covered by multiple probesets in a sample, the average gene probeset rank value was used [23].
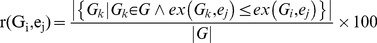
(1)where *ex(g,e)* is a function, which gives the mean normalized expression value of gene *g* in sample *e* and **G** is the set of all genes.

Computation of coefficient of variation value for each gene in each GEO dataset and identification of candidate reference genes

Let G =  {*G_1_*.....*G_m_*} be the set of genes and **S**  =  {*e_1_*.....*e_n_*} be a GEO dataset of *n* samples. For gene *G_i_* in experiment *e_j_*, a single rank value, *r*(*G_i_*.*e_j_*), was computed. Given a set ***S*** with *n* experiments, we had at most *n* rank values for a gene. The average amount of change in the rank of gene *G_i_*, in set ***S*** was then computed by the Coefficient of Variation CV(*S.G_i_*) as given below in Equation 2.
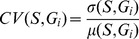
(2)where
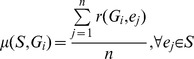
(3)and
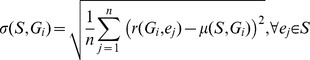
(4)


In this analysis, we computed a coefficient of variation value for each gene *G_i_* in each GEO dataset ***S***.

Next, we identified candidate reference genes with coefficient of variation values below a predefined threshold, *t*, observed in as many experiments as possible. In order to account for platform related differences, for each gene *G_i_* and threshold *t*, we computed a ratio. Ratio*_t_*(*G_i_*) as follows:
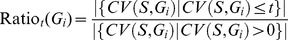
(5)


### Calculation of percentage of occurrence

Let *f*_PO_(*r*) be the function that gives the number of genes with *Ratio_t_* greater than a given *r* and occur in at least in *PO*% of the datasets (Equation 6).
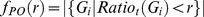
(6)


We plotted and compared the graph of *f*_PO_(*r*), varying *r* from 0 to 1, for housekeeping genes and randomly selected non-housekeeping genes.

### Calculation of specificity and sensitivity

We use the housekeeping gene set published by Eisenberg et al. [22] as the ground truth set of housekeeping genes. All the remaining genes are assumed to be non-housekeeping genes for the specificity and sensitivity analysis. The simple threshold classifier we use simply classifies all genes with a CV value below than a given threshold in at least Ratio*_t_* of the datasets as housekeeping genes. All these analyses are performed at a fixed percentage of occurrence of 75% and with a fixed Ratio*_t_* of 0.9. The ground truth genes that are identified as housekeeping genes by this classifier are true positives (TP) whereas, the ground truth genes that are identified as non-housekeeping genes are false negatives (FN). Similarly, genes not in the ground truth set but identified as housekeeping genes are false positive genes (FP) and genes not in the ground truth set identified as non-housekeeping genes by the classifier are true negatives (TN). Using these definitions, sensitivity is given by TP/(TP+FN) and specificity is computed as TN/(TN+FP).

### Cell Lines

Cell lines were obtained from the following sources and validated by STR analysis: HepG2 (ATCC HB-8065), FOCUS [35], Mahlavu[36], Hep3B (ATCC HB-8064), Hep3B-TR [37], Huh7 (JCRB JCRB0403), SkHep1 (ATCC HTB- 52), PLC (ATCC CRL-8024), MDA-MB-453 (ATCC HTB-131), HCC1937 (ATCC CRL-2336), BT-20 (ATCC HTB-19), T47D (ATCC HTB-133), CAMA- 1 (ATCC HTB-21), HCT116 (ATCC CCL-247), HT29 (ATCC HTB-38), SW620 (ATCC CCL-227).

### RNA extraction

RNA was extracted from 8 Hepatocellular Carcinoma cell lines (HepG2, FOCUS, Mahlavu, Hep3B, Hep3B-TR, Huh7, SkHep1, PLC), 5 Breast Carcinoma cell lines (MDA-MB453, HCC1937, BT20, T470, CAMA I) and 3 Colon Carcinoma cell lines (HCT116, HT29, SW620) with NucleoSpin Total RNA Isolation Kit and the concentration and purity of total RNA from each cell line was measured by using a NanoDrop Spectrophotometer (NanoDrop Technologies). Reverse transcription was performed using RevertAid First Strand cDNA Synthesis Kit (Fermentas) from 2 μg RNA with oligodT primer.

### Real-time quantitative PCR and stability analysis

Primers for 17 selected housekeeping genes were designed using the Primer3 software (Table S5 in Supporting Information S2). A cDNA dilution series for each primer set in triplicate was analyzed to calculate efficiencies of the primers using the linear regression slope of the dilution series with the equation Efficiency  =  10^(−1/slope)^-1. Real-time quantitative PCR assays were performed in duplicate for each candidate gene using 1 μl of 1∶100 diluted cDNA template with DyNAmo SYBR Green qPCR Kit (Finnzymes) on BioRad iCycler Real-Time qPCR System. The following program was used: Initial denaturation at 95°C 15 min amplification for 45 cycles (95°C 15 s followed by 57°C 30 s and 72°C 30 s), and final extension at 72°C 10 min. Melting curve analysis was done for each run, in addition to agarose gel electrophoresis, to confirm the amplicon size and presence of a single gene-specific peak free from any primer-dimer or genomic DNA amplification.

BioRad iCycler was programmed to set the Cq threshold on a fixed level. Cq values generated by BioRad iCycler system were transformed into quantities (relative expression values) according to Vandesompele *et al.* [25]. Relative expression values were calculated with the equation: Relative Expression  =  Efficiency^−ΔCq^. Coefficient of Variation (CV) was calculated as the ratio of standard deviation to the mean relative expression. geNorm and NormFinder software were used for stability analysis.

## Supporting Information

Supporting Information S1 **Reference Gene Lists.**
(ZIP)

Supporting Information S2 **Tables S1–S5.** Table S1. Kolmogorov–Simirnov Test results for the hypothesis comparing pair-wise equivalence of the ratio distribution of housekeeping gene set to the ratio distribution of random sets of genes excluding the housekeeping genes. Table S2. Kolmogorov–Simirnov Test results for the hypothesis comparing pair-wise equivalence of the ratio distribution of random sets of genes, excluding the housekeeping genes. Table S3. Stability values of 17 reference genes calculated by NormFinder and geNorm. Table S4. Stability values of 13 genes, with standard deviation lower than 1.42, calculated by NormFinder and geNorm. Table S5. Real-time quantitative PCR primers.
(PDF)

Supporting Information S3 **ROC Curves.**
(ZIP)
