# Supplementary material for: Identification of Novel Reference Genes Based on MeSH Categories
Source: PLoS One. 2014 Mar 28;9(3):e93341. doi: 10.1371/journal.pone.0093341 (PMC3969360; doi:10.1371/journal.pone.0093341)
Supplement: Supporting Information S2 — Tables S1–S5. Table S1. Kolmogorov–Simirnov Test results for the hypothesis comparing pair-wise equivalence of the ratio distribution of housekeeping gene set to the ratio distribution of random sets of genes excluding the housekeeping genes. Table S2. Kolmogorov–Simirnov Test results for the hypothesis comparing pair-wise equivalence of the ratio distribution of random sets of genes, excluding the housekeeping genes. Table S3. Stability values of 17 reference genes calculated by NormFinder and geNorm. Table S4. Stability values of 13 genes, with standard deviation lower than 1.42, calculated by NormFinder and geNorm. Table S5. Real-time quantitative PCR primers. (PDF) [file pone.0093341.s002.pdf]

Supporting Information

Table S1 Kolmogorov–Simirnov Test results for the hypothesis comparing pair-wise equivalence of the ratio distribution of housekeeping gene set to the ratio distribution of random sets of genes excluding the housekeeping genes. Random sets of genes had the same mean rank distribution as of housekeeping genes.

a)

House Keeping Gene Set vs. Random Sets*	CV ≤ 0.5		CV ≤ 0.1		CV ≤ 0.05		CV ≤ 0.01	
	D	p-value	D	p-value	D	p-value	D	p-value
Set 1	0.443	**<0.0001**	0.505	**<0.0001**	0.511	**<0.0001**	0.458	**<0.0001**
Set 2	0.454	**<0.0001**	0.484	**<0.0001**	0.493	**<0.0001**	0.463	**<0.0001**
Set 3	0.390	**<0.0001**	0.452	**<0.0001**	0.436	**<0.0001**	0.417	**<0.0001**
Set 4	0.417	**<0.0001**	0.481	**<0.0001**	0.479	**<0.0001**	0.424	**<0.0001**
Set 5	0.431	**<0.0001**	0.461	**<0.0001**	0.465	**<0.0001**	0.429	**<0.0001**

*Genes were observed at least 50% of the 381 GEO datasets (PO ≥ 50%).

b)

House Keeping Gene Set vs. Random Sets§	PO ≥ 75%		PO ≥ 50%		PO ≥ 25%		PO ≥ 5%	
	D	p-value	D	p-value	D	p-value	D	p-value
Set 1	0.385	**<0.0001**	0.511	**<0.0001**	0.587	**<0.0001**	0.555	**<0.0001**
Set 2	0.367	**<0.0001**	0.493	**<0.0001**	0.576	**<0.0001**	0.511	**<0.0001**
Set 3	0.352	**<0.0001**	0.436	**<0.0001**	0.518	**<0.0001**	0.500	**<0.0001**
Set 4	0.362	**<0.0001**	0.479	**<0.0001**	0.564	**<0.0001**	0.519	**<0.0001**
Set 5	0.348	**<0.0001**	0.465	**<0.0001**	0.544	**<0.0001**	0.512	**<0.0001**

§Genes had CV ≤ 0.05

Hypothesis:

H_0: The ratio distribution of housekeeping gene set and random set is not significantly different

H_a: The ratio distributions of the two samples are significantly different.

The bold font results in the table rejected the null hypothesis, H_0, as the computed p-values were lower than the significance level (p=0.05) in these cases.

Table S2 Kolmogorov–Simirnov Test results for the hypothesis comparing pair-wise equivalence of the ratio distribution of random sets of genes, excluding the housekeeping genes. Random sets of genes have the same mean rank distribution as of housekeeping genes.

a)

Random Set vs. Random Sets*	CV ≤ 0.5		CV ≤ 0.1		CV ≤ 0.05		CV ≤ 0.01	
	D	p-value	D	p-value	D	p-value	D	p-value
Set 1 vs. Set 2	0.049	0.357	0.034	0.769	0.041	0.571	0.030	0.761
Set 2 vs. Set 3	**0.127**	**0.000**	**0.104**	**0.003**	**0.102**	**0.004**	0.055	0.197
Set 3 vs. Set 4	0.088	0.019	0.071	0.091	0.074	0.067	0.025	0.919
Set 4 vs. Set 5	0.034	0.779	0.037	0.681	0.051	0.329	0.032	0.726

*Genes were observed at least 50% of the total sets (PO ≥ 50%).

b)

Random Set vs. Random Sets §	PO ≥ 75%		PO ≥ 50%		P O≥ 25%		PO ≥ 5%	
	D	p-value	D	p-value	D	p-value	D	p-value
Set 1 vs. Set 2	0.042	0.243	0.041	0.571	0.042	0.606	0.083	**0.039**
Set 2 vs. Set 3	0.172	0.172	0.102	**0.004**	0.005	**0.005**	0.053	**0.039**
Set 3 vs. Set 4	0.076	0.076	0.074	0.067	0.022	**0.022**	0.057	0.319
Set 4 vs. Set 5	0.032	0.529	0.051	0.329	0.352	0.352	0.053	0.393

§Genes had CV ≤ 0.05 to the number of sets the gene is observed.

Hypothesis:

H_0: The ratio distributions of random sets are not significantly different

H_a: The ratio distributions of the two sets are significantly different.

The bold font results in the table rejected the null hypothesis, H_0, as the computed p-values were lower than the significance level (p=0.05) in these cases.

Table S3 Stability values of 17 reference genes calculated by NormFinder and geNorm.

Gene	NormFinder				geNorm			
	All	Liver	Breast	Colon	All	Liver	Breast	Colon
**RPS10**	0.25	0.19	0.42	0.12	0.00	0.00	0.00	0.00
**RPL41**	0.26	0.29	0.29	0.11	0.00	0.00	0.00	0.00
**RPL7**	0.26	0.19	0.29	0.20	0.27	0.22	0.25	0.28
**RPS3A**	0.23	0.23	0.11	0.07	0.34	0.30	0.38	0.13
**RPL30**	0.21	0.11	0.26	0.22	0.37	0.31	0.31	0.25
**HBXIP**	0.34	0.29	0.33	0.29	0.44	0.45	0.17	0.20
**CFL1**	0.30	0.29	0.18	0.35	0.47	0.42	0.35	0.46
**RPS17**	0.39	0.19	0.49	0.38	0.51	0.27	0.50	0.31
**EEF2**	0.43	0.38	0.30	0.70	0.55	0.53	0.43	0.65
**ACTB**	0.48	0.43	0.24	0.32	0.60	0.50	0.47	0.41
**GAPDH**	0.39	0.25	0.62	0.39	0.64	0.38	0.78	0.52
**H2AFZ**	0.49	0.33	0.64	0.25	0.68	0.48	0.82	0.17
**AARS**	0.60	0.60	0.73	0.28	0.72	0.58	0.66	0.49
**TPT1**	0.63	0.72	0.47	0.38	0.76	0.72	0.55	0.35
**SOD1**	0.63	0.75	0.62	0.47	0.80	0.65	0.72	0.59
**RPN2**	0.73	0.78	0.29	0.51	0.85	0.79	0.59	0.56
**GSTO1**	1.02	1.17	1.16	0.43	0.94	0.91	0.93	0.38

Table S4 Stability values of 13 genes, with standard deviation lower than 1.42, calculated by NormFinder and geNorm.

	NormFinder				geNorm			
	**All**	**Liver**	**Breast**	**Colon**	**All**	**Liver**	**Breast**	**Colon**
**RPL30**	0.22	0.19	0.23	0.19	0.37	0.31	0.31	0.25
**RPS3A**	0.23	0.31	0.04	0.07	0.34	0.30	0.38	0.13
**RPL7**	0.25	0.22	0.31	0.19	0.27	0.22	0.25	0.28
**RPS10**	0.25	0.21	0.41	0.09	0.00	0.00	0.00	0.00
**RPL41**	0.28	0.37	0.22	0.12	0.00	0.00	0.00	0.00
**CFL1**	0.30	0.22	0.24	0.39	0.47	0.42	0.35	0.46
**HBXIP**	0.31	0.25	0.31	0.24	0.44	0.45	0.17	0.20
**RPS17**	0.41	0.26	0.52	0.34	0.51	0.27	0.50	0.31
**GAPDH**	0.43	0.21	0.68	0.45	0.64	0.38	0.78	0.52
**ACTB**	0.48	0.36	0.28	0.32	0.60	0.50	0.47	0.41
**H2AFZ**	0.52	0.32	0.68	0.19	0.67	0.48	0.82	0.17
**AARS**	0.57	0.57	0.66	0.35	0.72	0.58	0.66	0.49
**SOD1**	0.63	0.73	0.59	0.50	0.80	0.65	0.72	0.56

Table S5 Real-time quantitative PCR primers

**Gene**	**Primer**	**Oligo sequence (5'→3')**	**Amplicon size (bp)**
AARS	sense	CCAGTGGCAGAAGGATGAAT	206
	antisense	GCTTCAAGGCTTCATTCAGG	
ACTB	sense	GGACTTCGAGCAAGAGATGG	234
	antisense	AGCACTGTGTTGGCGTACAG	
CFL1	sense	GTGCTCTTCTGCCTGAGTGA	247
	antisense	TCTTCTTGATGGCGTCCTTG	
EEF2	sense	AACGGCAAGTTCAGCAAGTC	182
	antisense	TTGTCCTTGTCCTCGCTGTC	
GAPDH	sense	GGCTGAGAACGGGAAGCTTGTCAT	272
	antisense	CAGCCTTCTCCATGGTGGTGAAGA	
GSTO1	sense	TCGATCCGCATCTACAGCAT	271
	antisense	TTCCAGAACTGGCACCAGAC	
H2AFZ	sense	GTGCGACGAAGGAGTAGGTG	176
	antisense	CTCTGCGGTGAGGTACTCCA	
HBXIP	sense	GCAGCACTTGGAAGACACAA	294
	antisense	TCCTATGACAGGCTGCTGAA	
RPL30	sense	CCTAAGGCAGGAAGATGGTG	171
	antisense	GGCAGTTGTTAGCGAGAATG	
RPL41	sense	AAGATGAGGCAGAGGTCCAA	248
	antisense	TCCAGAATGTCACAGGTCCA	
RPL7	sense	AAGGTGTTGCAGCTTCTTCG	164
	antisense	TTGCCATAACCACGCTTGTA	
RPN2	sense	GCTCTCGCATAATCGCTACC	289
	antisense	CCGGTTGTCACCTTCAACTT	
RPS10	sense	CGCAGAGATGTTGATGCCTA	162
	antisense	AGAGACTGCATGGCCTTCAT	
RPS17	sense	CAACGACTTCCACACGAACA	210
	antisense	ATCTCCTGATCCAAGGCTGA	
RPS3A	sense	CCGATGGTTACTTGCTTCGT	193
	antisense	CCAATGCTGTCTGGAATCAAT	
SOD1	sense	AGGCTGTACCAGTGCAGGTC	193
	antisense	ATGATGCAATGGTCTCCTGA	
TPT1	sense	ATGAATCCAGATGGCATGGT	167
	antisense	TGTGGATGACAAGCAGAAGC	
